# Generation and characterization of temperature-sensitive alleles encoding GPI anchored proteins Psu1 and Dfg502 in *Schizosaccharomyces pombe*

**DOI:** 10.17912/micropub.biology.001179

**Published:** 2024-04-02

**Authors:** Bita Tavafoghi, Liping Ren, Kathleen L. Gould, Alaina H. Willet

**Affiliations:** 1 Cell and Developmental Biology, Vanderbilt University School of Medicine, Nashville, TN, US

## Abstract

Glycosyl-phosphatidylinositol (GPI) anchored proteins are implicated in remodeling of the yeast cell wall during growth and division. *Schizosaccharomyces pombe* proteins, Psu1 , Dfg501 , and Dfg502 are predicted GPI anchored proteins with likely cell wall modifying activity. Here, we isolated and characterized null and temperature-sensitive alleles that will allow further analysis of the function of these proteins and *S. pombe* cell wall formation. Our data confirm that Psu1 is necessary for cell separation, maintaining proper cell shape, and viability. Additionally, we found that Dfg501 and Dfg502 share a redundant and essential function necessary for cell separation and viability.

**Figure 1. Characterization of psu1 and dfg502 mutant alleles:**
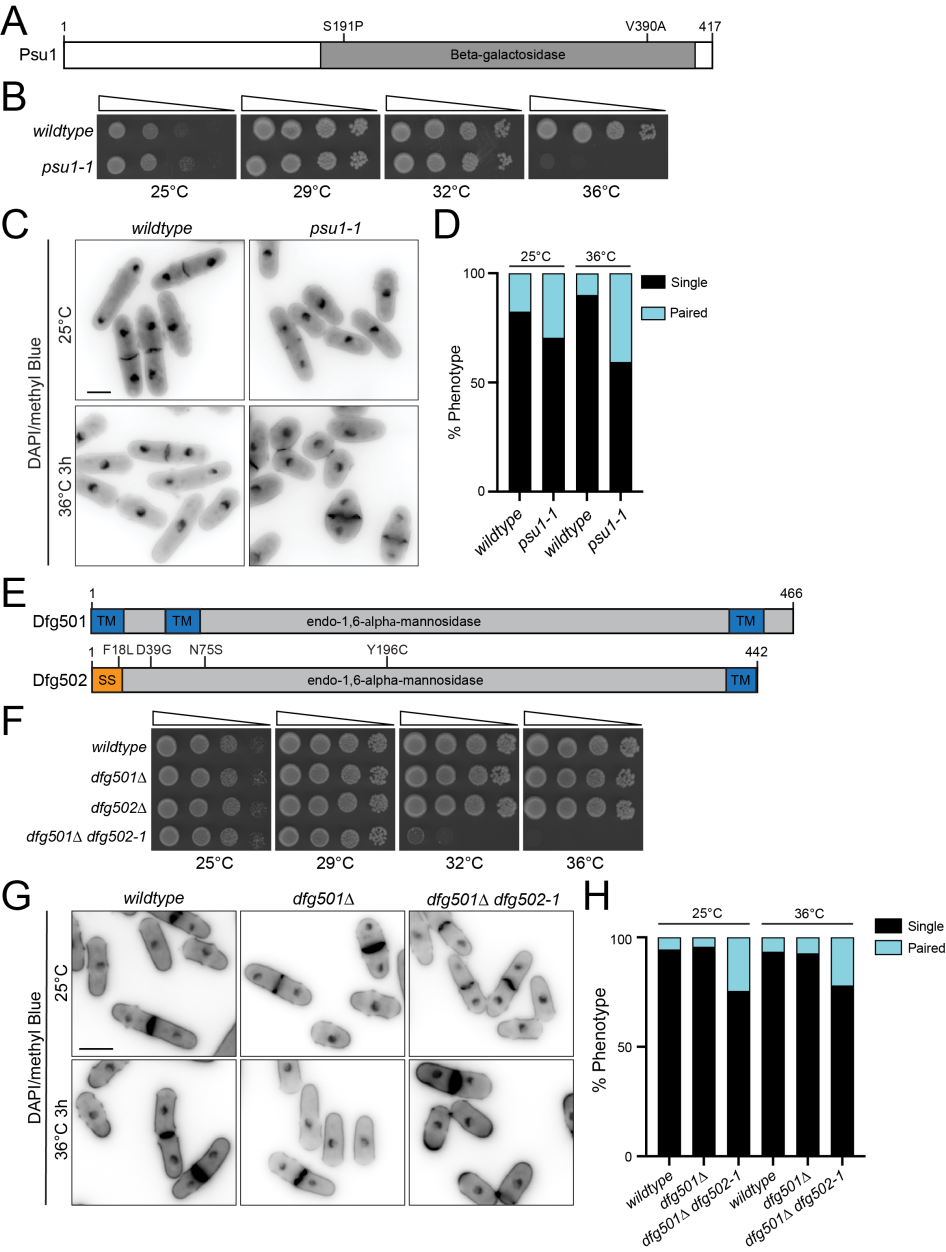
(A) A schematic, drawn to scale, of the protein encoded by *psu1* . The amino acid substitutions in *psu1-1* are indicated. (B, F) The indicated strains were grown in liquid YE at 25°C until they reached mid-log phase. Then, 10-fold serial dilutions were made and 2.5 µL of each was spotted on YE agar plates and incubated at the indicated temperatures for 3 days prior to imaging. (C, G) The indicated strains were grown at 25°C and then shifted to 36°C for 3 hours prior to fixation with 70% ethanol. Samples were taken at each timepoint. Cells were washed three times with PBS and then stained with methyl blue (MB) and DAPI prior to imaging. (D, H) The indicated phenotypes were quantified from the same experiment as in C or G. n ≥ 330 for each. Paired cells have not completed cell separation. (E) A schematic, drawn to scale, of the proteins encoded by *dfg501* and *dfg502* . The amino acid substitutions in *dfg502-1* are indicated. Signal sequence (SS), transmembrane domain (TM).

## Description

Cell-walled organisms build and remodel their cell wall during growth and division. The *Schizosaccharomyces pombe* cell wall is mainly composed of linear and branched glucan chains and galactomannan (Bush et al., 1974; Humbel et al., 2001; Osumi et al., 1998) . Additionally, the cell wall contains a cohort of proteins linked directly to it (de Groot et al., 2007) . Many of these are modified with a glycosyl-phosphatidylinositol (GPI) anchor and established to play important roles in the structure and function of the yeast cell wall (De Groot et al., 2003; Pittet & Conzelmann, 2007) . To become GPI-anchored, proteins are modified on a ω site near their C-terminus by lipid addition in the endoplasmic reticulum (Frieman & Cormack, 2004) . The lipid anchor can either be cleaved at the plasma membrane or remodeled (Fankhauser et al., 1993; Martin-Yken et al., 2001) . The specific way that GPI-anchored proteins are attached to the cell surface might be critical for maintaining cell wall homeostasis and allow cells to adapt to different environmental stresses. The function of GPI modified proteins is not fully understood. Here, we isolated and characterized a temperature-sensitive allele of *psu1* . We also generated null alleles of the adjacent and presumably duplicated *dfg501* and *dfg502* genes and a temperature-sensitive *dfg502* allele in the *dfg501∆* strain.

Psu1 contains a beta glucosidase domain belonging to the glycosidase hydrolase 132 (GH132) enzyme family (Drula et al., 2022; Gastebois et al., 2013) . Other fungal members of this enzyme class hydrolyze linear β-(1,3)-glucans (Gastebois et al., 2013) and are implicated in promoting cell separation and septum integrity (Mouassite et al., 2000; Ritch et al., 2010) . *S. pombe* *psu1* is essential and when deleted the cells display cell separation defects, become rounded, and eventually lyse and die (Omi et al., 1999) .

To further study Psu1 function, we isolated a new *psu1* temperature-sensitive allele using an error-prone PCR method (Tang et al., 2019) . Sequencing revealed substitutions of serine 191 to proline and valine 390 to alanine within the catalytic domain ( Figure 1A ). Serial dilutions of wildtype and *psu1-1* cells spotted at a variety of temperatures showed that both strains grew similarly at 25°C, 29°C and 32°C, but *psu1-1* did not grow at 36°C while wildtype grew robustly ( Figure 1B ). We next grew cells at 25°C, shifted some of them to 36°C for 3 hours, and then fixed the cells from each condition. The cells were stained with DAPI and methyl blue (MB) to mark DNA and the cell wall, respectively, and imaged. We found that at 25°C *psu1-1* cells had an increase in paired cells compared to wildtype and this difference was exacerbated at 36°C, indicating a cell separation defect ( Figure 1C ). Additionally, consistent with the *psu1∆* phenotype, *psu1-1* cells were rounded at high temperature ( Figure 1C ) (Omi et al., 1999) . Thus, the temperature-sensitive and null mutant phenotypes are concordant, and our data validates the importance of Psu1 for maintaining proper cell shape and promoting cell separation following cytokinesis (Omi et al., 1999) .

*dfg501* and *dfg502* encode putative endo-1,6-α-mannosidases belonging to the glycoside hydrolase 76 (GH76) enzyme family (Drula et al., 2022) and are orthologous to *Saccharomyces cerevisiae* Dfg5 (Kitagaki et al., 2002) . Endo-1,6-α-mannosidases hydrolyze unbranched 1,6-α-mannose and these enzymes are hypothesized to have a role in transferring GPI anchored proteins from the plasma membrane to the cell wall, however their precise function has not been confirmed biochemically (Kitagaki et al., 2002) .

To study *dfg501* and *dfg502* function we aimed to make single and double gene deletions of these adjacent genes. While we were able to construct *dfg501∆* and *dfg502∆* single deletion strains, we were unable to recover a double deletion strain. We therefore generated a *dfg502* temperature-sensitive allele in a *dfg501∆* genetic background. The temperature-sensitive allele contained F18L, D39G, N75S and Y196C substitutions within Dfg502 ( Figure 1D ). Analysis of cell growth revealed that each single deletion grew similarly to wildtype at all temperatures tested but *dfg501∆ dfg502-1* had reduced growth at 32°C compared to wildtype and did not grow at 36°C ( Figure 1E ). Fixing and staining of the cells with DAPI and MB revealed that *dfg501∆ dfg502-1* cells had an increase in the frequency of paired cells at both 25°C and 36°C compared to wildtype cells, indicative of a cell separation defect ( Figure 1F ). These results indicate that Dfg501 and Dfg502 are important for promoting cell separation, which may be a common theme among GPI anchored enzymes. Interestingly, *gas1-1* , *psu1-1* and *dfg501∆ dfg502-1* all have cell separation defects (Howard et al., 2024) . Dfg5 and Gas1 defective cells also have similar phenotypes noted in *S. cerevisiae* , suggesting they may play similar roles in remodeling the cell walls of these two yeast species (Kitagaki et al., 2002) .

In conclusion, GPI anchored proteins are physically poised to enact cell wall modification to allow for cell growth and division and it will be interesting to learn how they collaborate in space and time to allow precise cell wall remodeling in response to cell growth and environmental changes.

## Methods

Yeast methods

*S. pombe* strains were grown in yeast extract (YE) and standard *S. pombe* mating, sporulation, and tetrad dissection techniques were used to construct new strains (Moreno et al., 1991) .

Molecular biology methods

The *psu1* allele was sequenced by generating a PCR product with an oligonucleotide 85 bp upstream of the start site (CTTCGTTCGTTCCTTGAATTTTAGACACA) and a reverse oligonucleotide within *kanMX6* (Integrated DNA technologies). The PCR product was sequenced with a forward oligonucleotide 580 bp into the open reading frame (GGTGATGGTGCTGGTTCCTCTTGCGTTG) and a reverse oligonucleotide 679 bp into the open reading frame (GCAAACCACCACGGGTTTCACCGTCAG).

The *dfg502-1* allele was sequences by generating a PCR product with an oligonucleotide 300 bp upstream of the start site (GCTCGCATTGAAATTTATTTGGTTAC) and a reverse oligonucleotide within *kanMX6* (Integrated DNA technologies). The PCR product was sequenced with a forward oligonucleotide 144 bp into the open reading frame (CCATAAATAGTGCCTTGACTACCGTCACTGACGG) and a reverse oligonucleotide 860 bp into the open reading frame (CCTTCCATATGGGTTTGCCACACAGAACTGCC). The *dfg501* and *dfg502* gene deletions were made as previously described (Chen et al., 2015) .

Isolation of temperature sensitive alleles with error-prone PCR

Temperature-sensitive mutants of *psu1* and *dfg502* were constructed and isolated based on the previously described protocol (Tang et al., 2019) but using EX taq polymerase (Takara, 4025) and accompanying dNTPs (Takara, RR01BM). The *dfg502* temperature sensitive allele was made in the *dfg501 ::kanMX6* genetic background and *hphMX6* selection linked to *dfg502* .

Microscopy and image analysis

Strains for fixed-cell imaging experiments were grown at 25°C in YE and then shifted to 36°C for 3 hours. Cells were fixed with 70% ethanol for DAPI and methyl blue (MB) staining as described previously (Roberts-Galbraith et al., 2009) . Images were acquired using a Zeiss Axio Observer inverted epifluorescence microscope with Zeiss 63× oil (1.46 NA) and captured using Zeiss ZEN 3.0 (Blue edition) software. A singular medial Z slice was obtained. All images were further processed using ImageJ (Schindelin et al., 2012) . Graphs were constructed with Prism 8.0 (GraphPad Software).

## Reagents

The strains used in this study and their genotypes are listed below.

**Strain** **Genotype** **Source**

KGY246 *ade6-M210 leu1-32 ura4-D18 h ^-^* Lab stock

KGY5216-2 *psu1-1(S191, V390A):kanMX6 ade6-M210 leu1-32* This study

*ura4-D18 h ^-^*

KGY3698-2 *dfg501Δ::kanMX6* *ade6-M210 leu1-32 ura4-D18* *h ^-^* This study

KGY3699-2 *dfg502Δ::kanMX6* *ade6-M210 leu1-32 ura4-D18 h ^-^* This study

KGY5820-2 *dfg501Δ::kanMX6* *ade6-M210 leu1-32 ura4-D18* *h ^-^* This study

*dfg502-1(F18L, D39G, N75S, Y196C):hphMX6*
